# American Dental Society of Europe*Read before the Forty-second Annual Meeting, held in London, England, July 28, 1922.

**Published:** 1923-01

**Authors:** W. A. Spring

**Affiliations:** New York City


### AMERICAN DENTAL SOCIETY OF EUROPE*

### President's Address

BY W. A. SPRING, D. D. S., OF NEW YORK CITY

*Fellow Members and Guests of the American Dental Society of Europe:*

After eight years, filled with tremendous changes in the lives of most of us, due to the terrible World War, I greet you again. When our retiring President told us an hour after midnight in that banquet hall in Paris that the French Army was mobilizing, little did we dream what the future held in store for us. Some of us have been through sorrow, all of us have been through anxiety and trials, all of us have suffered losses, though it is also true that some of us have been forced into our blessings.

Whatever the various experiences in our lives have been, I am sure that the old fires of brotherly affection, so characteristic of the members of our society, have not died out. No, on the contrary, they are burning brighter than ever today. The fact that so many years of our lives have been spent three thousand miles away from our own people and our own land, banded together in a common cause, has no doubt contributed to the affection we bear each other.

What a joy it is to come together today! It is with great personal pleasure that I find myself again in dear old Europe and among the friends who have meant so much to me. Our joy today, however, is mingled with sadness, for our ranks have been thinned by terrible losses. The names of Jenkins, Davenport, Younger, Daball, Bogue, Burt, Crane, Foerster, Bryan, Patton, Cooper, Larabee, Law and others have passed into history.

---

* Read before the Forty-second Annual Meeting, held in London, England, July 28, 1922.

We must close up the ranks and stand firmer and closer together, utilize our entire resources in membership and increase our numbers in order not to feel too keenly our losses.

The American Dental Society of Europe is entering upon the fiftieth year of its existence. Forty-nine years ago on the Fourth of July, our society was founded on the summit of the Rigi, surely as patriotic a service, I am sure, as any group of men could perform on such a day. The twenty-fifth anniversary of our society was celebrated by a wonderful, never-to-be-forgotten meeting here in London, and it seems eminently fitting that this renewal of our life, after lying dormant for eight years, should be made right here. We rejoice that we can exist again as an active society, and we all approach it with alacrity and vigor. That is, indeed, well, for we have a big task confronting us. Our society has always stood for the best, and the proceedings of our meetings have constituted a true record of the very foremost of dental thought. We recall with pride the scientific wisdom of Miller, the golden words of Jenkins, the sound thought and good judgment of Kirk Davenport, not to mention the achievements of the living.

To live up to our reputation and, moreover, to make up for eight years of inactivity we have real work ahead and much to record. A history of our society and its proceedings is a history of dentistry during the years of our activity. No good and great thing in our profession has failed to give an ample reading in the ever active pulse of our society. It is also true that the incubi in the blood stream of professional and dental progress have been fought with commendable vigor.

We, indeed, find ourselves with a reputation to live up to. The burden is greater due to our years of inactivity, but we are young and virile yet and shrink not, and shall therefore be equal to the task.

Allow me briefly to review some of the developments of the past eight years.

DEVELOPMENTS IN DENTISTRY IN PAST EIGHT YEARS

*First:* And most conspicuous has been the progress in oral surgery, much of it due directly to the war, and many splendid results have been accomplished by direct co-operation between the general and the dental surgeon. Reports of it have been made from time to time, but we must not fail to present and spread on our minutes a fitting record of this work. We are, in fact, the more proud to do so, because members of our society have held first place in these truly marvellous achievements.

*Second:* Great progress has been made in the general use of the X-ray in dental practice during the past eight years. A limited number of progressive men have used it for years—Morton, Kells Van Woert, Rhein, Ottolengui and Gillette were pioneers in this work. Rhein had used it in 1901, and with diagnostic wires in root canals in 1905. Our own Dr. Abbott read a paper on it before our society at Wiesbaden in 1909, and yet in 1914 the number of dental radiographers was very limited.

During these years the changes have been nothing short of marvellous. In 1914, 1915 and 1916, many of our profession, some of them conspicuously prominent and successful, fought against it. Some quoted their successes in past years without it. They apparently did not want to see too distinctly and be compelled to adopt a new technique requiring so many hours of labor. Today some kind of an X-ray machine is universally considered an essential part of dental equipment. Dentists no longer have to plead with patients for the privilege of taking a picture nor apologize for requiring it as an aid to diagnosis, but are even importuned to take more pictures. This great change has increased our usefulness, raised our profession to a higher plane, and has made life safer for humanity.

*Third:* There has come about a great change in the attitude of the dental and medical professions, or perhaps better said, a sharpening of our mental vision, on the subject of focal infections. Our ideal is no higher than before. We have always believed in perfect root canal fillings and perfect health at the apices of all teeth, but the X-ray has given us glasses, has almost removed cataracts from our eyes, and a vast vision of diseased areas is now staring us in the face and fairly burning into our consciousness. Some of us have found the first year with an X-ray machine a nightmare, with hideous dreams of black areas and of diagnostic wires, frequently sticking through the sides of the root. It has inaugurated a new era, and compelled a new and improved technique and more accurate fillings, and incidentally shown that many teeth formerly retained were a menace. We are now infinitely better able to live up to our ideal of completely filled canals. We have been so quickened by our own scientific workers, and the attitude of the medical profession, that we are more alert than ever before to remove a tooth which is not or cannot be made perfectly sound. Mere absence of pain has ceased to lull us to contentment.

The attention of the world is now focussed upon the teeth. It is our duty and privilege to know more about the teeth and contiguous parts than anyone else, and to constitute the court of last appeal. This brings with it a tremendous responsibility. The insidious danger of infection in the blood stream should never be lost sight of. We hold the lives of our patients in our hands as truly, if not as conspicuously, as the physician and surgeon.

However, he who extracts a tooth just because of the loss of the pulp is brutal. Millions of pulpless teeth are healthy. We have plenty of evidence of pulpless teeth which have even been badly diseased and are now healthy with regenerated bone at the apex.

We must use judgment, and remove without hesitation, teeth of unfavorable or uncertain prognosis. We should be especially prompt in action with patients in ill health or lowered resistance, who cannot afford the risk of infection.

*Fourth:* Root resection has made tremendous progress in the last eight years. Many a pulpless tooth has lost pericementum at the apex, which renders complete restoration to health, to say the least, doubtful. The X-ray does not show it, but an area which fails to fill in with bone after proper sterilization and complete canal filling is pretty good evidence of it. Thousands of such teeth are being restored to sound health today by root end resection. The skillful operator makes a generous incision to the bone, lifts the periosteum and flap of gum, removes enough process to amply expose the diseased part, and removes it. Subsequent examination of these areas have proved perfect health, in fact, the success of this operation. The percentage of success is high, and every dentist should either familiarize himself with the operation, or be on the alert to send his patients to some one who performs it.

*Fifth:* Removable bridgework. While it is true that the removable bridge *per se* is not a development of the past eight years, improvements and refinements and its almost universal adoption have been conspicuous. Eight years ago a few experts were using removable bridges, while the great majority of dentists were using fixed bridges or none at all. Today most dentists are believers and users of some kind of a removable bridge, and the man who has the courage to advocate a fixed bridge is rare indeed. The movable, removable bridge was in its infancy eight years ago, and has had a marvellous development and a well deserved success. The slogan of Dr. Chayes, its discovered, that "teeth move in function," though universally recognized, is not followed by everyone to what Chayes considers the logical conclusion, that all bridges should be made movable. It so happens that Ash and Peeso removable, though rigid bridges, have records of many years of service, even as high as thirty. Therefore, a concensus of opinion exists that the particular form of attachment which best fits the case can be chosen with freedom. Dr. Doxtater has developed some very small though powerful attachments for removable bridges, which are especially advantageous because teeth with living pulps may be readily utilized for removable bridge work without danger to the pulps.

A cast clasp restoration developed by Dr. Nesbett, and presented to the profession in 1916, has great value in some places and obviates the necessity of mutilating the abutment teeth. It is known as the Nesbett bridge.

The record of this meeting, on the various kinds of removable bridges, will be worthy of the traditions of the society.

*Sixth:* The attitude of the medical and dental professions to each other has undergone a great change during the past eight years. Dentistry will never be just a specialty of medicine. It is in some respects more than that already. An ever increasing knowledge of medicine is to our advantage, and is being utilized. It is giving us a broader grasp and is no doubt partly responsible for greater co-operation. It is, in fact, increasingly apparent that co-operation is becoming firmly established between the two professions, and that is well, for only in that way can the best good of the patient be achieved. During the war, your President had charge of the Dental Department of the Dispensary at the Presbyterian Hospital in New York, and the deference of the medical men in that institution was a good indication of the present attitude of the medical profession.

At the annual meeting of the New York State Dental Society, held in the City of New York in 1921, one of the most interesting and valuable features was a symposium in which dentists and physicians took part in discussing "Dental Disease as Related to Systemic Disease." The cordial attitude of the two professions to each other was conspicuous. Prof. Sydney R. Miller of Johns Hopkins, was especially happy in his remarks. After speaking of the personal debt which every physician owes to the dental profession, for the institution and maintenance of a Dental Research Fund, so enthusiastically and universally supported, he says: "From its studies, information of inestimable value has come from both the clinical and laboratory side. This information has been stimulating and helpful alike to dentists and physicians, with one result among others, that there is a more universal recognition of a far too little appreciated fact—the medical and dental professions are not fundamentally separate, but rather branches of one art and science which has for its aim the prevention and alleviation of human suffering."

A joint meeting of physicians and dentists was held this year at the Academy of Medicine in New York, presided over conjointly by the President of the Academy of Medicine and the President of the First District Dental Society of New York. The President of the Academy of Medicine exhibited a feeling of cordiality and admiration for our profession, and the keynote of the meeting was the recognized need of each profession for the other, and the advantage to humanity of hearty co-operation. It is our duty and privilege to occupy just as important a position today in the great healing art, as the medical profession. To a great extent they look to us to occupy it and expect it of us. Let us accomplish our destiny to the full.

Nearly three years have passed since the great war, and yet we stand today only on the threshold of reconstruction. Nations like individuals need each other. Any nation to be prosperous must have business and trade relations with other nations, who are sufficiently prosperous to buy, and pay their obligations and in return to produce the things which we require. These are fundamental principles. Business men are ignoring the many animosities which were increased and multiplied by the war and are trying to establish, as universally as possible, a business *modus vivendi.*

A great opportunity presents itself to us, members of a great profession, devoted to the alleviation of human suffering to be leaders in the great work of reconstruction. We all know how nobly surgeons worked to save lives and prevent suffering for friend and foe alike on both sides of the firing line. Let us, therefore, now forget all differences arising from birth, race or politics and practice the Golden Rule.

A common ground, our profession, to which we have devoted our lives, invites co-operation. Let us welcome with real cordiality members of our profession from all countries. Thus only can we set an example to the world of real and true greatness. Thus can we do our real share towards helping this poor old world back to a condition of normalcy.—*Dental Items of Interest.*

